# Bioinspired seeding of biomaterials using three dimensional microtissues induces chondrogenic stem cell differentiation and cartilage formation under growth factor free conditions

**DOI:** 10.1038/srep36011

**Published:** 2016-11-03

**Authors:** J. Leijten, L. S. Moreira Teixeira, J. Bolander, W. Ji, B. Vanspauwen, J. Lammertyn, J. Schrooten, F. P. Luyten

**Affiliations:** 1 Prometheus, Division of Skeletal Tissue Engineering, KU Leuven, Leuven, Belgium; 2 Skeletal Biology and Engineering Research Center, Department of Development and Regeneration, KU Leuven, Leuven, Belgium; 3 Department of Developmental BioEngineering, MIRA Institute for Biomedical Technology and Technical Medicine, University of Twente, Enschede, The Netherlands; 4 BIOSYST-MeBioS, Department of Chemistry, KU Leuven, Leuven, Belgium

## Abstract

Cell laden biomaterials are archetypically seeded with individual cells and steered into the desired behavior using exogenous stimuli to control growth and differentiation. In contrast, direct cell-cell contact is instructive and even essential for natural tissue formation. Namely, microaggregation and condensation of mesenchymal progenitor cells triggers chondrogenesis and thereby drives limb formation. Yet a biomimetic strategy translating this approach into a cell laden biomaterial-based therapy has remained largely unexplored. Here, we integrate the microenvironment of cellular condensation into biomaterials by encapsulating microaggregates of a hundred human periosteum-derived stem cells. This resulted in decreased stemness-related markers, up regulation of chondrogenic genes and improved *in vivo* cartilage tissue formation, as compared to single cell seeded biomaterials. Importantly, even in the absence of exogenous growth factors, the microaggregate laden hydrogels outperformed conventional single cell laden hydrogels containing supraphysiological levels of the chondrogenic growth factor TGFB. Overall, the bioinspired seeding strategy described herein represents an efficient and growth factor-free approach to efficiently steer cell fate and drive tissue formation for biomaterial-based tissue engineering strategies.

The proficiency with which chondrogenic differentiation is induced in multipotent stem cells directly affects the outcome of cell laden biomaterial based skeletal tissue engineering strategies. In recent years, a plethora of studies have focussed on the modification of biomaterials with biomimetic elements such as proteins or peptides, *in vitro* pre-treatments of implants, controlled release of chondrogenic growth factors, co-culturing distinct cell types and even genetic modification of cells^1^,^2^,^3^,^4^,^5^,^6^,^7^. In essence, all mentioned approaches attempt to create a microenvironment instructive for improved cartilage formation. Yet nature’s own developmental mechanism of microaggregating progenitor cells to generate a chondrogenic microenvironment has remained largely unexplored in the field of cell laden biomaterials.

Microaggregation and condensation of progenitor cells is a key event that drives chondrogenesis in early limb bud development via the creation of a unique microenvironment^8^. The importance of this knowledge has long since been recognized and incorporated into golden standard cell culture models such as micromasses of ~200.000 cells^9^,^10^,^11^,^12^. However, no biomimetic strategy for cell laden biomaterials has been developed to capitalize on this well-known phenomenon. Specifically, the encapsulation of micromasses in biomaterials is problematic due to the creation of vast cell free areas within the biomaterial. As a result, biomaterials such as hydrogels archetypically contain a population of individual cells, which is a less chondrogenically potent formulation. We hypothesize that seeding biomaterials with cellular microaggregates of a few dozen cells, instead of dispersed progenitor cells, will enable cell specification and subsequent augmentation of the implants’ chondrogenic capacity. Moreover, a microaggregate based approach will allow for a more homogenous cell seeding within the biomaterial as compared to the conventional micromasses. However, it has remained largely unknown if microaggregates of a few dozen cells behave distinctly from micromasses of ~200.000 cells.

We recently have reported on the development of a high throughput platform for highly controlled production of cellular microaggregates of 50 to 250 cells^13^. Importantly, this platform enables the facile production of high quantities of stem cell microaggregates, which can be incorporated within the biomaterials using standard single cell seeding techniques.

Herein, we report on the effects of microaggregating human periosteum-derived progenitor cells on chondrogenic differentiation and cartilage formation both *in vitro* and *in vivo*. In particular, we demonstrate that microaggregation can be used as an easy biomimetic stimulatory pre-treatment prior to induce cartilage formation in a growth factor free manner.

## Materials and Methods

### Microwell fabrication

A patterned silicon wafer was fabricated using standard soft lithographic techniques. In short, a custom designed mask was fabricated using a 25.000 DPI photoplot printer (Koenen, Germany). This mask was used to photo-pattern SU-8 photoresist on top of a silicon wafer. The design consisted of an array of 125.000 circles, each with a diameter of 200 micrometer and an inter-circle space of 100 micrometer (Supplemental Fig. 1). The circles were patterned on top of the silicon wafer with controlled heights of 66 or 150 micrometers by varying the centrifugal speed of the SU-8 spin coater. Using replica molding, a master mold of micropatterned polydimethylsiloxane (Sylgard 184, Dow Corning) was fabricated. Using the mold, disks of 3% agarose microwell were fabricated for non-adherent cell culture. Inserts of 1.5 cm were punched out of the disks using a sterile biopsy puncher, placed in 24 well culture plates and sterilized using ultra-violet light for 30 minutes. This method allowed for high throughput production of microaggregates with controlled cell density per aggregate.

### Cell culture and microaggregate formation

Human periosteum derived stem cells (hPDCs) are mesenchymal stem cells that can be isolated from the periosteum. These cells play an important role in fracture healing by rapidly producing large quantities of cartilage to stabilize the fracture. Indeed, isolated hPDCs display excellent chondrogenic differentiation capacity. For this study, hPDCs were isolated from six donors (age 14.9 ± 2.1, 3 male and 3 female) as previously described^14^. All isolated hPDCs were pooled and expanded in DMEM containing 10% of fetal bovine serum up to passage five, and used for experimentation. All donors were individually validated on their chondrogenic potential. Cells were seeded at 5.000 cells per cm^2^ and subcultured directly prior to reaching confluence. Subsequently, to minimize unpredictable serum effects, cells were starved for 24 hours in DMEM containing 0,1% fetal bovine serum followed by a seven days preconditioning period in serum-free medium^15^. Subsequently, the cells were detached using TripLE (Life Technologies), counted, washed and reseeded in a 24 well plate containing a microwell insert. To create aggregates composed of 50, 100 or 250 cells, the microwells were covered in 2 ml of serum free medium containing 100.000, 200.000 or 500.000 cells, respectively. Microaggregates were cultured up to 21 days in microwells in either the presence or absence of 10 ng/ml of TGFB1 (Peprotech), and used for *in vitro* analysis. Thrice a week 1,5 ml of the medium was refreshed.

### Flow cytometry

hPDCs cultured in monolayer or aggregates were characterized for expression of stemness markers (CD73, CD90, and CD105) by flow cytometry using human MSC Phenotyping kit (Lot# 130-095-198, Miltenyi Biotec, NL). hPDCs were dispersed using TripLE (Life Technologies), suspended in a flow cytometry staining buffer solution (eBioscience Inc.,USA, Lot#E00015-1639), and stained in accordance to manufacturer’s instructions. In brief, 100 μl of cell suspension (up to 1 × 10^6^) was mixed with 10 μl of MSC Phenotyping Cocktail and incubated for 10 minutes without light at 4 °C. Subsequently, hPDCs were washed and analyzed using BD FACS CantoTM using the cell analyzer (BD Biosciences, San Jose, CA) and FlowJo V10 software.

### Scanning Electron Microscopy

Microwells were chemically dehydrated using graded ethanol and hexamethyldisilazane, coated with 5 nm of palladium and platinum blend and imaged using a scanning electron microscope (Philips XL40) equipped with a lanthanum hexaboride electron gun.

### Gene expression analysis

Total RNA was isolated using an RNeasy mini kit (Qiagen) and measured using a Nanodrop ND2000 (Thermo Scientific). Complementary DNA (cDNA) was synthesized using the RevertAid H Minus First Strand cDNA Synthesis Kit (Thermo Scientific) and 500 ng of non-amplified total RNA. For each condition a total of 20 ng of cDNA was amplified using a Fast Sybr green master mix (Applied Biosciences) and a Corbett rotor gene QPCR (Qiagen). All steps were performed according to their respective manufacturer’s instructions. Gene expression was normalized on beta-actin (*ACTB*) expression. ACTB was validated to act as a stable reference gene within the experiments dataset. Primer sequences can be found in table 1. Three biological replicates were carried out per time-point.

### Glycosaminoglycan and collagen deposition

Microaggregates were cultured in microwells and compared to an equal amount of single cells after being cultured for up to 21 days under chondrogenic conditions. Glycosaminoglycans (GAGs) were quantified using a glycosaminoglycan assay kit (Biocolor) following manufacturer’s instructions (n = 3). Collagen deposition was quantified using a hydroxyproline colorimetric assay kit (Biovision) using standard operating procedures (n = 3). DNA content was measured using Quant-iT^TM^ PicoGreen^®^ dsDNA assay (Invitrogen, Merelbeke, Belgium) according to the manufacturer’s instructions (n = 3).

### Histological evaluation

All samples were fixed using 4% paraformaldehyde for 1 hour. To allow easy handling, the *in vitro* microaggregates for histological purposes were embedded in 2,5% agarose (Invitrogen). All samples were dehydrated, embedded in paraffin, cut into 5 micrometer section using a microtome (Microm HM360 Prosan) and stained using histology and immunohistochemistry unless otherwise stated. To visualize glycosaminoglycans samples were stained with acidic Alcian Blue (pH = 1, Merck) and counterstained with nuclear fast red (Vector Laboratories). General cell morphology was visualized using hematoxyline (SigmaAldrich) and eosin staining (Klinipath). To visualize general tissue morphology and highlight circulating erythrocytes sections were stained with Masson’s Trichrome (SigmaAldrich). Apoptotic cells were visualized using terminal deoxynucleotidyl transferase (TdT) dUTP nick-end labeling (TUNEL) assay, which was combined with DAPI counterstaining to visualize all cells. Histological sections were microphotographed (X83P22F, Olympus). Semi-quantification was achieved with semi-automated analysis using ImageJ software.

### *In vivo* performance

All the procedures on animals were approved by KU Leuven’s ethical committee for Animal Research. The animals were housed according to the guidelines of the Animalium Leuven (KU Leuven). All procedures were carried out in accordance with the approved guidelines. Eight-week-old, female NMRI nu^−/−^ mice were maintained in isolator cages in pathogen-free conditions. Microaggregates of 100 cells were cultured for 6 days in microwells in either the presence or absence of 10 ng/ml of TGFB1. Subsequently, the microaggregates were washed with PBS and incorporated in semihemispherical 100 μl hydrogels of collagen type I (5 mg/mL, BD Biosciences) with a cell density of 10 million cells per ml. Single cell seeded gels were used as controls, keeping the same cell density per ml. After gelation, the constructs were implanted subcutaneously into the back of anesthetized nude mice. One and three weeks post implantation, the mice were sacrificed and the samples were excised. All samples were fixated using 4% paraformaldehyde for 1 hour at room temperature and processed for histologic and immunohistochemical evaluation. For each condition and time point six individual replicates were implanted and evaluated.

### Immunohistochemical evaluation

Paraffin embedded sections were deparaffinised in histoclear, hydrated in methanol and stained for transcription factors sex determining region Y-box 9 (SOX9) and collagen 2 (COL2). For SOX9, the specimens were incubated SOX9 antibody (1:200, Rabbit polyclonal, NovusBiologicals) with Biotinylated SP-conjugated goat anti-rabbit (1:500), Streptavidin Alexa 555 (1:500) (Jackson ImmunoResearch) and DAPI (1:2500, Sigma Aldrich). For COL2, the specimen were incubated with pepsin (1 mg/mL in 5mM HCl, Sigma Aldrich), 3% H2O2 (Chem-LAB), SOX9 antibody (1:200, MAB 8887, Millipore), goat anti mouse (1:500, Jackson ImmunoResearch), 3,3′-diaminobenzidine substrate (DAKO) and hematoxyline (SigmaAldrich). Specimen were mounted in Mowiol for confocal microscopic analysis using an Olympus FluoView FV1000 and visualized by the samples by Z-stacking 35 images of 26.22 micrometer. SOX9 nuclear translocation was artisanally semi-quantified by measuring whether DAPI positive nuclei were SOX9 positive using Image J software. For each condition 300 cells were counted.

### Statistical analysis

Each experiment was performed in triplicate unless otherwise specified. The results are presented as mean ± standard deviation (SD). Experimental data were analyzed for statistical significance using a student t-test or one way ANOVA where appropriate. Statistical significance was indicated as follows: a p-value ≤ 0.05 with * or ^#^, ≤ 0.01 with ** or ^##^ and ≤ 0.001 with *** or ^###^.

## Results

### Controlled high throughput generation of stem cell microaggregates

**Figure 1:**
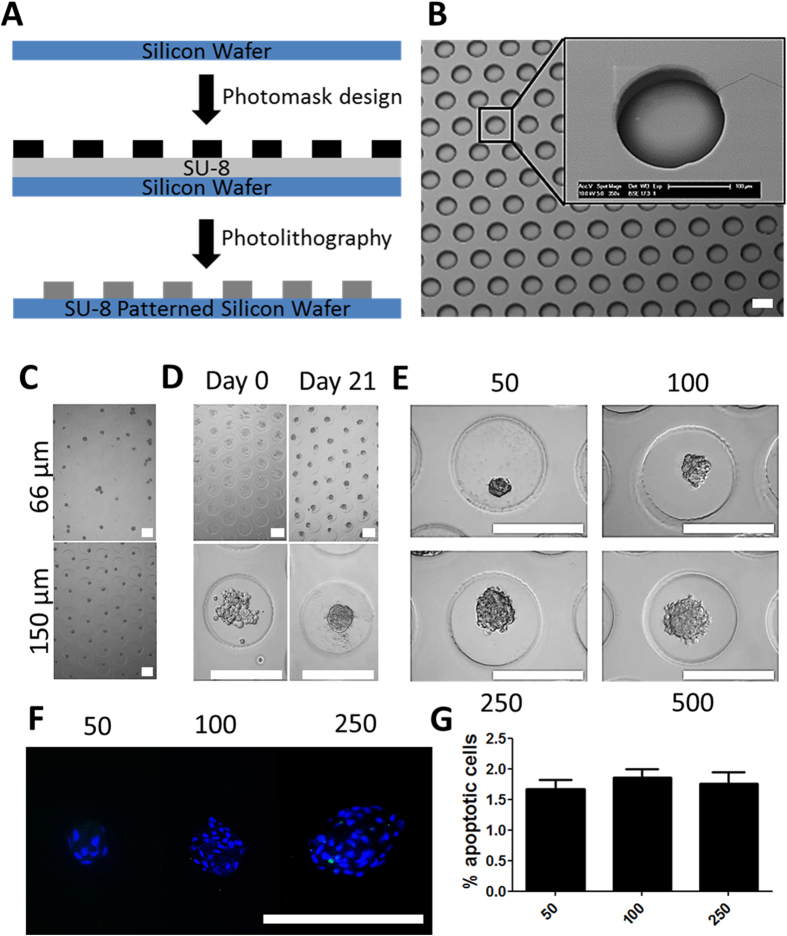
Development of the high throughput microaggregation platform. (**A**) Photolithography was used to pattern micropillars on a silicon wafer that acts as a master mold. (**B**) SEM microphotograph of an agarose cast of the master mold demonstrated high quality arrays of microwells. (**C**) Human periosteum derived stem cells were seeded in microwells with a depth of either 66 or 150 micrometer (**D**) and allowed for at least 21 days of culture. (**E**) By varying the cell seeding densities microaggregates of 50 to 500 cells could be generated with a high degree of control. To determine the percentage of apoptotic cells, microaggregates were stained using (**F**) terminal deoxynucleotidyl transferase dUTP nick end labeling, which was (**G**) semi-quantified. Scale bars equal 200 μm.

Using photolithography, a silicon wafer was micropatterned with an epoxy-based negative photoresist (Fig. 1A). The wafer’s design was composed of an array of 125.000 perfect circles with a diameter of 200 micrometer (Supplemental Fig. 1A). Circles were spaced 100 micrometers apart. Then a negative copy was created using polydimethylsiloxane, which served as a master mold to cast 3 percent agarose disks, which enables a microfabricated non-adherent surface for the culture of thousands of aggregates. Using biopsy punchers microwell inserts fitting standard culture plates were obtained (Supplemental Fig. 1B). A 24 well microwell insert contains 2000 individual microwells. Scanning electron microscopy demonstrated the high fidelity nature of the procedure’s pattern transfer, which resulted in near perfected circles with slightly rounded bottoms (Fig. 1B). Human periosteal stem cells were seeded onto microwell inserts with preselected heights ranging from 50 to 250 micrometers. Light microscopical analysis revealed that a depth of 150 micrometer was optimal to generate microaggregates and allow their subsequent *in vitro* culture (Fig. 1C). In particular, microwells with less depth allowed for microaggregate formation, but did not prevent trans-microwell migration of the newly formed microtissues. In contrast, microwells with a depth of 150 micrometer, or more, retained the microaggregates for at least up to 21 days in culture (Fig. 1D). By varying the cell seeding density we were able to generate microaggregates of various sizes. Specifically, microaggregates containing 50 to 500 cells can be reproducibly produced, cultured and collected for analysis (Fig. 1E). Microaggregates composed of a higher amount of cells were successfully generated, but could not be cultured without additional handling steps as it depleted the medium’s nutrients in less than 24 hours. High cell survival in microaggregates of 50, 100, and 250 cells was validated by revealing that only ~1.5% of the cells underwent apoptosis after seven days of culture (Fig. 1F,G).

### Microaggregation reduces expression stemness markers

**Figure 2:**
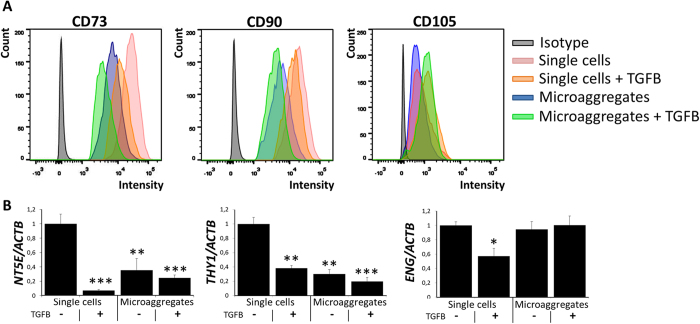
Microaggregation and TGFB reduce stemness markers expression. (**A**) Single or microaggregated hPDCs cultured for seven days in either the presence or absence of 10 ng/ml of TGFB were analyzed on CD73, CD90 and CD105 using FACS and (**B**) on the expression of their respective genes *NTE5*, *THY1* and *ENG* using RT-qPCR. Data represent the mean of three donors, each measured in quadruplicate ± SD. *P < 0.05; **P < 0.01; ***P < 0.001.

Microaggregation provides stem cells with a condensating three dimensional microenvironment that is reminiscent of the developing limb bud. A key feature of the chondrogenic commitment of limb bud stem cells is a decrease in their stemness markers. Indeed, exposure of TGFB to periosteal stem cells decreased cell surface expression of CD73 and CD90 by ~3 and ~2 fold, respectively. Interestingly, microaggregation more potently decreased the surface expression of these biomarkers than TGFB by ~6 and 7 fold, respectively. Moreover, combining microaggregation and TGFB exposure further decreased the expression CD73 and CD90 by 10 and 9 fold, respectively (Fig. 2A). This effect was at least in part explained by changes of mRNA levels as both TGFB and microaggregation result in lower expression levels of the genes *NT5E* and *THY1*, which encode CD73 and CD90 respectively (Fig. 2B). Microaggregation did not affect the cell surface expression of CD105 nor the mRNA expression levels of its encoding gene *ENG*. This might be explained by the relatively low basal expression level of CD105 in the used periosteal stem cells.

### Microaggregates allows for TGFB induced SOX9 nuclear translocation

**Figure 3:**
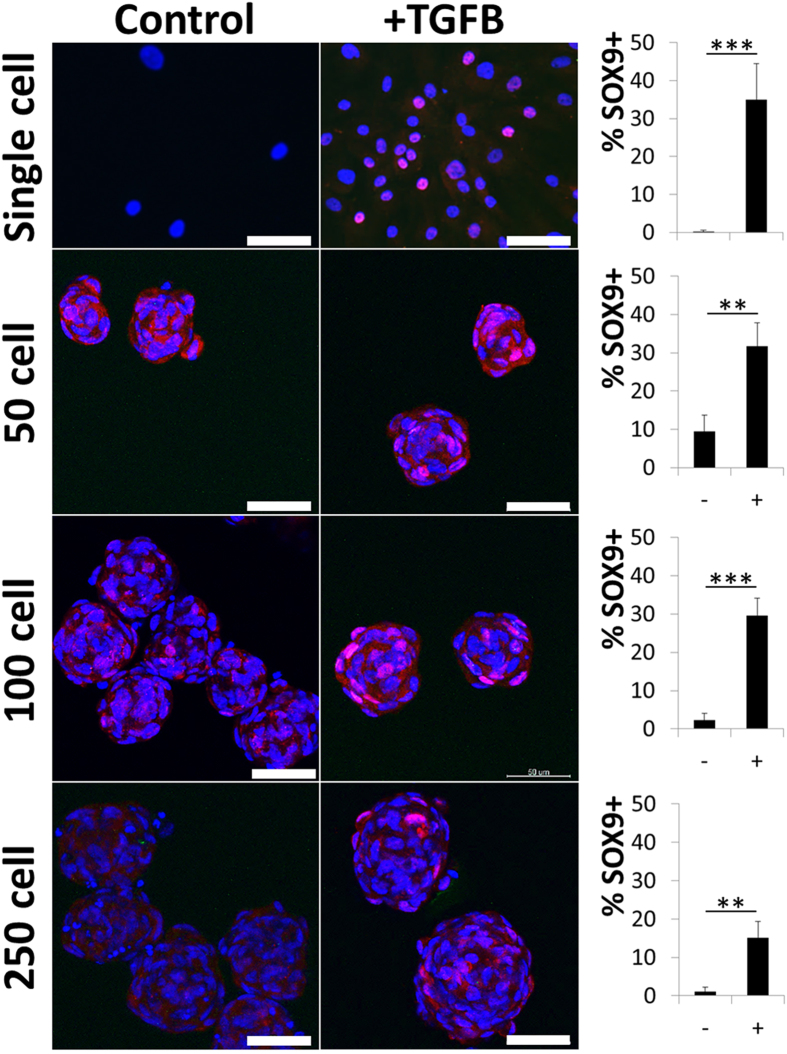
SOX9 nuclear translocation in single and microaggregated hPDCs. hPDCs cultured as single cells or microaggregates for seven days in either the presence or absence of 10 ng/ml of TGFB were immunohistochemically stained for SOX9, microphotographed and semi-quantified. Scale bars equal 200 μm. **P < 0.01; ***P < 0.001

The high cell density and compact three dimensional nature of the microaggregates could potentially slow down, or even prevent, the diffusion of exogenously added growth factors to the core of the cellular construct. To investigate this, we exposed microaggregates composed of 50, 100 or 250 cells to TGFB and compared their SOX9 nuclear translocation to those of conventional monolayers. To this end, microaggregates were successfully imaged from top to bottom using fluorescent confocal microscopy and represented as stacked images (Fig. 3). SOX9 mediated transcription is a key event in chondrogenesis. Microaggregates of 50 and 100 cells demonstrated a similar percentage of cells with SOX9 positive nucleus, which ranged between 30 and 35 percent. It is of note that a fraction of the cells in the microaggregates were noticeably more intensely stained as compared to their monolayer counterparts. 50 cell microaggregates demonstrated SOX9 positive cells throughout the microtissues. In contrast, 100 cell microaggregates had a slight preference for the more peripheral region and less so for the microtissue’s core. Furthermore, microaggregates of 250 cells had a significantly lower fraction of cells demonstrating SOX9 nuclear translocation (~17%), which were typically located in the more peripheral regions. In mono layer cultures without TGFB the percentage of SOX9 positive cells was negligible. Strikingly, microaggregated cultures without TGFB contained up to 10% percent SOX9 positive cells. This phenomenon was microaggregate-size dependent with higher percentages of SOX9 positive cells as well as more intensely stained cells in smaller microaggregates.

### Microaggregation improves chondrogenic transcriptional profile

**Figure 4:**
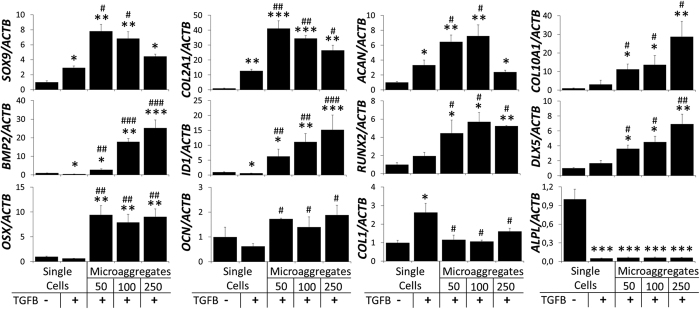
Microaggregation induces a chondrogenic gene expression fingerprint. hPDCs were cultured as single cells or microaggregates in the presence or absence of 10 ng/ml of TGFB for a period of seven days and analyzed on gene expression using RT-qPCR. *P < 0.05; **P < 0.01; ***P < 0.001 as compared to single hPDCs without TGFB. ^#^P < 0.05; ^##^P < 0.01; ^###^P < 0.001 as compared to single hPDCs with TGFB.

**Figure 5:**
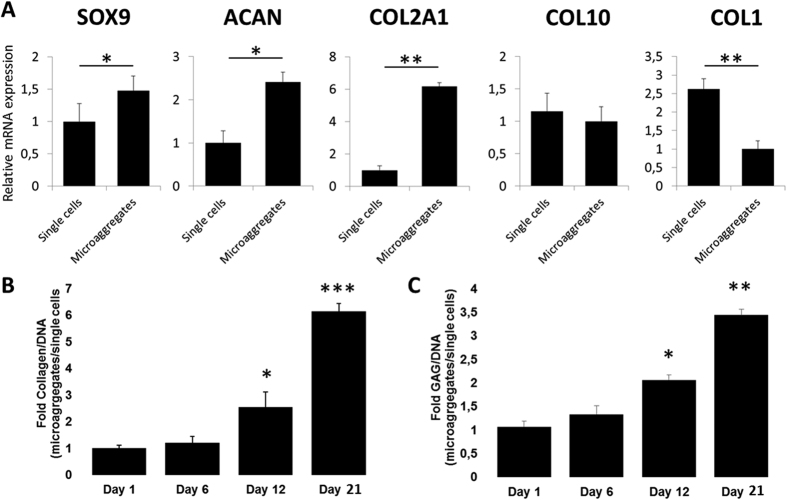
Microaggregation results in an improved chondrogenic gene expression fingerprint after three weeks in culture. hPDCs were cultured as single cells or microaggregates of 100 cells for a period of 21 days and analyzed on their respective gene expression of *SOX9*, *ACAN*, *COL2A1*, *COL1A1* and *COLX*. Moreover, their (**B**) collagen and (**C**) glycosaminoglycan (GAG) were measured on various time points and normalized to their DNA content, which demonstrated significant elevations in collagen and GAG production. Data is represented as mean ± SD. N = 4. *P < 0.05; **P < 0.01.

To fingerprint the effects of microaggregation on the chondrogenic differentiation of periosteal stem cell mRNA levels were quantified after 6 days of culture (Fig. 4). Matching the SOX9 immunohistochemistry, supplementation of TGFB and microaggregation, in particular using 50 and 100 cells, increased *SOX9*, *COL2A1* and *ACAN* mRNA levels. *COL10A1* mRNA increased slightly with TGFB and more strongly with microaggregation. Although TGFB is by far the most commonly used growth factor to induce chondrogenic differentiation, we have previous reported that *in vivo* mouse periosteal stem cells are driven into the chondrogenic lineage by bone morphogenetic protein (BMP)2^16^. Interestingly, while TGFB supplementation decreased *BMP2* mRNA expression in single cells, microaggregation potently increased *BMP2* mRNA levels in a microaggregate-size dependent manner. To investigate if this had a measurable downstream effect we measured the BMP direct target gene *ID1* mRNA levels, which tightly followed BMP2 expression. BMP2 induced osteochondral differentiation operates via RUNX2 and is mediated via DLX5. The mRNA levels of these two genes were not significantly changed by TGFB supplementation, but potently increased upon microaggregation regardless of their size. Moreover, microaggregation, but not TGFB supplementation, increased *OSX* and *OCN* mRNA levels. Interestingly, *COL1* mRNA expression levels were increased by TGFB exposure in the single cell control, yet this was nullified upon microaggregation. In addition, TGFB supplementation strongly decreased *ALPL* mRNA expression, which was not further augmented by microaggregation. Furthermore, to ensure that the improved chondrogenic gene expression profile was continued over time, single cells and microaggregates of 100 cells were analyzed on gene expression after being cultured in the presence of TGFB for 21 days (Fig. 5A). Indeed, as compared to single cells, microaggregation increased *SOX9* (~1,5 fold), *ACAN* (~2,5 fold), and *COL2A1* (~6 fold), decreased *COL1* (~2,5 fold) and did not change *COL10A1* mRNA expression levels. This gene expression pattern is indicative that microaggregation is more conducive to chondrogenic differentiation that single cells. In addition, we determined that collagen deposition became significantly higher in microaggregates as compared single cells being ~2,5 and 6,5 fold higher after 12 and 21 days of culture (Fig. 5B). Similarly, GAG deposition was significantly higher in microaggregates than single cells by ~2 and 3,5 fold after 12 and 21 days of culture (Fig. 5C).

### Microaggregation improves *in vivo* cartilage formation

We then investigated whether microaggregation also improved *in vivo* chondrogenesis and subsequent cartilage formation. To this end, human periosteal cells cultured as either single cells or microaggregates in presence or absence of TGFB for 6 days were loaded into a collagen hydrogel.

**Figure 6:**
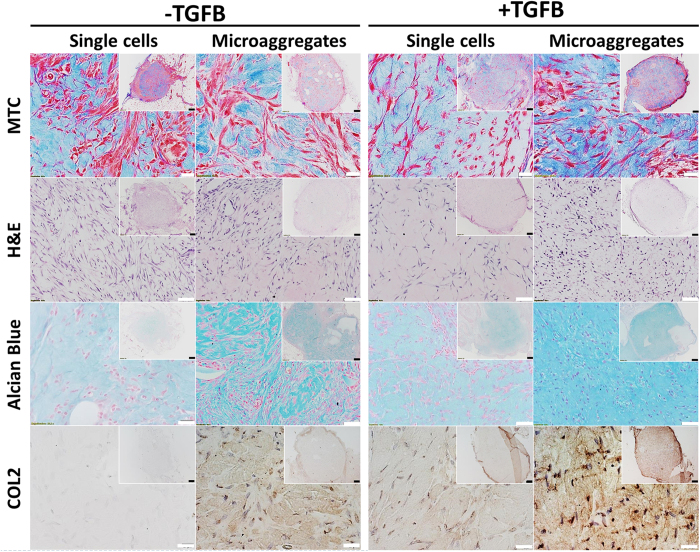
Cartilage formation in implants containing either single or microaggregated hPDCs one week post implantation. After six days of *in vitro* culture, hPDCs were laden in 5 mg/ml collagen hydrogels, subcutaneously implanted for one week and analyzed histologically to visualize the implant and invading perfused blood vessels using Masson’s trichrome, cellular morphology using hematoxylin and eosin and cartilage formation using Alcian blue and COL2 (n = 6). White scale bars equal 200 μm. Black scale bars equal 1 mm.

For these experiments, microaggregates of 100 cells were selected based their on their overall stability and chondrogenic behavior. At this time point, no detectable levels of glycosaminoglycans were present in any of the conditions (Supplemental Fig. 2). Consequently, any detectable levels of glycosaminoglycans will be solely deposited during the *in vivo* phase. One week post implantation samples were retrieved and sectioned (Fig. 6).

Masson’s trichrome allowed for identification of the implant by staining the collagen hydrogel. Moreover, this staining revealed that all samples of the single cells without TGFB were invaded with numerous small blood vessels, even in the core. This was less observed in the implants that contained microaggregates and not observed in cells exposed to TGFB. Interestingly, within this short time frame, the microaggregates largely disassembled into single cells that migrated homogeneously throughout the entire implant. This could potentially be explained by the cell’s ability to potently bind to the biomaterial, which could stimulate the disassembly of the encapsulated microaggregates. These observations were corroborated using H&E staining and underlined the generally spindle shaped morphology of the cells in all implants.

Masson’s trichrome staining revealed numerous perfused blood vessels that were solely present in the single cell implants that were not exposed to TGFB. In addition, several blood vessels were observed around, but not within, the microaggregate implant without TGFB. In contrast, no blood vessels were observed in samples containing TGFB. Moreover, the collagenous matrix in which the single cells without TGFB were encapsulated stained noticeably less intense than any other implant. This potentially indicated matrix remodeling, which would correlate with its intense vascular invasion. This was corroborated by H&E staining, which revealed numerous vessels and fat-like cells within the implants. In contrast, the other specimen shared a distinct morphology of rounded cells located within matrix, phenotypically reminiscent of chondrocytes. The small fissures within the matrix are likely to be caused by cellular change of the original spindle shaped cells into a more round morphology.

**Figure 7:**
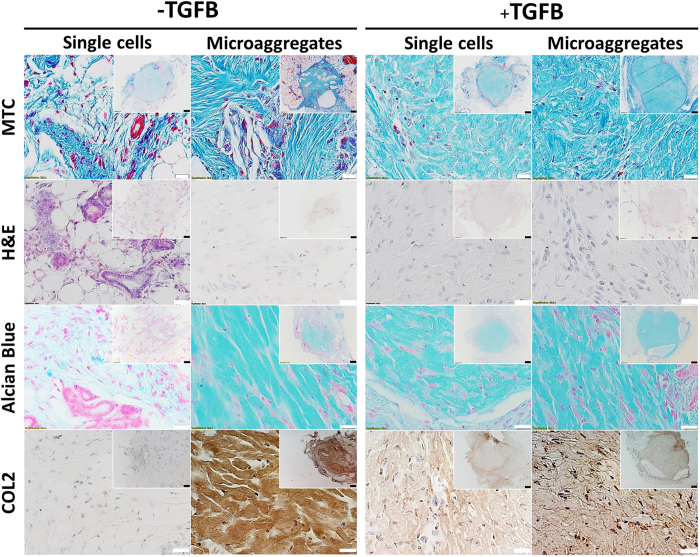
Cartilage formation in implants containing either single or microaggregated hPDCs three weeks post implantation. After six days of *in vitro* culture, hPDCs were laden in 5 mg/ml collagen hydrogels, subcutaneously implanted for three weeks and analyzed histologically to visualize the implant and invading perfused blood vessels using Masson’s trichrome, cellular morphology using hematoxylin and eosin and cartilage formation using Alcian blue and COL2 (n = 6). White scale bars equal 200 μm. Black scale bars equal 1 mm.

As expected, single cell without TGFB stimulation deposited only marginal amounts of glycacosaminoglycans and COL2, whereas TGFB supplementation boosted their deposition. However, as is typical of stimulated one week old implants, the overall levels of deposited glycosaminoglycans and COL2 remained low. In contrast, microaggregation of the periosteal stem cells resulted in strikingly higher levels of deposited glycosaminoglycans and COL2, even in the absence of TGFB. At three weeks post implantation, single cells that did not receive TGFB supplementation did not demonstrate any positive staining for glycosaminoglycans and COL2, while implanted that did receive TGFB supplementation demonstrated a modest level of staining. Regardless, implants that were originally microaggregated stained more intensely for COL2. Importantly, microaggregated hPDCs that did were not exposed to TGFB demonstrated the most intense COL2 staining. Together our data suggests that seeding biomaterials with chondrogenic microenvironments as provided by the cellular microaggregates outperforms conventional growth factor loading from the perspective of cartilage formation (Fig. 7).

## Discussion

Natural tissues are constantly exposed to a spatiotemporally mediated array of cell-cell interactions. In fact, their function depends on it. Even in mature tissues where the cells are imprisoned within extracellular matrix without any direct cellular contact, cell-cell interactions are important for their development. For example, cellular condensation is essential to the formation of a cartilaginous anlage during limb development. Moreover, chondrocytes form cellular clusters in osteoarthritis and are considered as a regenerative response^13^. Although these phenomena are widely known, they have as of yet not been translated into a biomimetic strategy to augment stem cell based tissue engineering strategies. The purpose of this work was to develop and explore a high-throughput stem cell microaggregation system to seed biomaterials with the biomimetic element of condensation. We demonstrated that with this approach one can steer cell fate and boost cell differentiation without the need of expensive recombinant growth and differentiation factors and time consuming *in vitro* protocols.

Human periosteal stem cells generate large amounts of de novo cartilage and bone during a bone fracture^17^. As such they represent a promising cell source for cartilage and bone tissue engineering. Moreover, during the initial phases of fracture healing, the periosteal stem cells are in direct contact with other periosteal cells. When creating a tissue engineered implant to heal complicated fractures or bone trauma, cells are typically laden into a biomaterial such as a hydrogel^18^,^19^. In contrast to the natural healing response, the encapsulated stem cells are archetyped as single cells without cell-cell contact. However, whether this is detrimental to the regenerative response, neotissue formation and successful healing has remained an open question. In this study we revealed that forcing human periosteal stem cells into microaggregates greatly improved their cartilage forming ability. As the microaggregates of 50 to 250 cells are relatively small, we observed much lower levels of apoptosis than those of the traditional micromasses of 200.000, which can suffer from nutrient diffusion limitation. Most notably, microaggregation quickly reduced stemness markers, which could suggest the initiation of lineage specification. Indeed, we have observed that microaggregation intensified SOX9 nuclear staining and correlated with increased expression of genes that are driven by SOX9 mediated transcription. Although these correlations match our observations, the mechanism driving this SOX9 mediated chondrogenesis remains unknown. Multiple possible explanations include effects of direct cell-cell communication, altered biomechanical stimulation, increased local concentration of growth factors and/or nutrient competition. Moreover, due to the increase in surface area-to-volume ratio it can lead to substantially enhanced mass transport in the microaggregates.

A key observation in this study was the rapid *in vivo* cartilage formation in the biomaterial implants containing microaggregates, even when no growth factors were supplemented. Conventionally, growth factors have been considered as a cornerstone of inducing tissue formation. TGFB in particular is considered a golden standard as a potent inducer of chondrogenesis. Chondrogenesis of stem cells still relies on the use of supraphysiological doses of this highly expensive recombinant protein. This has been reported to cause serious adverse effects including synovial hyperplasia and chondron-osteophyte formation^20^,^21^. Thus the development of methods that enable TGFB free chondrogenesis such as microaggregate induced chondrogenesis can potentially be of high clinical value. Based on histological analysis, microaggregation elicits a more rapid and intense cartilage formation than TGFB. In consequence, microaggregation can be considered an interesting and viable alternative for growth and differentiation factors to induce chondrogenesis in progenitor cells and might prove more cost-effective while potentially reducing adverse effects. Moreover, growth factors rarely have a single effect. In fact, when using supraphysiological dosages the occurrence of unexpected or even unwanted behavior in neighboring tissues is likely. For example, in our study TGFB was used to induce chondrogenesis in stem cells. However, implants containing untreated periosteal stem cells demonstrated rapid and intense vascularization, while TGFB supplemented implants prevented the infiltration of any blood vessels. Although this might be advantageous when creating articular cartilage, inhibition of angiogenesis is detrimental for the formation of endochondral bone. It is of note that the currently presented *in vivo* data have been based on a subcutaneous model, and not a critical defect model. As such, further research using an orthotopic model in an immunocompetent model might benefit our current understanding of the *in vivo* behavior of our implants.

An anticipated drawback of the local concentration of cells via microaggregation was the inherent consequence of the presence of larger cell free areas within the biomaterial. This might cause tissue inhomogeneity due to gradient wise matrix deposition and concomitant biomechanical instability of the implant. However, within the first week the microaggregated stem cells dissembled and dispersed near homogeneously throughout the implant. This is likely explained by the balance between cell-cell and cell-biomaterial interaction. The collagen hydrogel provides binding motifs for the encapsulated cells’ integrin receptors allowing for migration out of the microaggregate. As such, the choice of biomaterials and the incorporation of cell binding motifs can determine the speed of microaggregate disassembly, if at all present. This approach might prove viable to create improved migration or chemotactic assays, which incorporates the 3D nature of tissue nodules such as small tumors^22^. Regardless, while the microaggregates disassembled *in vivo*, they maintained their therapeutic benefit and resulted in an implant that combines the matrix homogeneity of single cells with the improved matrix formation of microaggregated cells.

## Conclusions

Collectively, our data supports that biomimicking the chondoinductive process of progenitor condensation can be achieved through microaggregation of stem cells. Notably, microaggregation can be used as an efficient treatment to steer cell fate, from naïve progenitor behavior towards a chondrogenic phenotype. As compared to single cells, biomaterials containing microaggregated periosteal stem cells demonstrated rapid and intense cartilage formation, both *in vitro* and *in vivo*. In short, this biomimetic seeding strategy represents a novel growth factor free approach boosting the performance of biomaterial based implants.

## Additional Information

**How to cite this article**: Leijten, J. *et al*. Bioinspired seeding of biomaterials using three dimensional microtissues induces chondrogenic stem cell differentiation and cartilage formation under growth factor free conditions. *Sci. Rep.* **6**, 36011; doi: 10.1038/srep36011 (2016).

**Publisher’s note**: Springer Nature remains neutral with regard to jurisdictional claims in published maps and institutional affiliations.

## Supplementary Material

Supplementary Information

## Figures and Tables

**Table 1:** qPCR primers used in this study.

Gene	Forward primer	Reverse primer	Temperature
*ACAN*	5′-AGGCAGCGTGATCCTTACC-3′	5′-GGCCTCTCCAGTCTCATTCTC-3'	60 °C
*ACTB*	5′-CCCAGATCATGTTTGAGACCT-3'	5′-CCTCGTAGATGGGCACAGT-3'	60 °C
*ALPL*	5′-GCTTCAAACCGAGATACAAGCA-3'	5′-GCTCGAAGAGACCCAATAGGTAGT-3'	60 °C
*BMP2*	5′-ACTACCAGAAACGAGTGGGAA-3'	5′-GCATCTGTTCTCGGAAAACCT-3'	60 °C
*COL1*	5′-GACGAAGACATCCCACCAAT-3'	5′-AGATCACGTCATCGCACAAC-3'	60 °C
*COL2A1*	5′-GGCTTCCATTTCAGCTATGG-3'	5′-AGCTGCTTCGTCCAGATAGC-3'	60 °C
*COL10A1*	5′-ACGATACCAAATGCCCACAG-3'	5′-GTGGACCAGGAGTACCTTGC-3'	60 °C
*DLX5*	5′-CAGCCAAAGCTTATGCCGAC-3'	5′-CGGTCACTTCTTTCTCTGGCT-3'	60 °C
*ENG*	5′-CACTAGCCAGGTCTCGAAGG-3'	5′-CTGAGGACCAGAAGCACCTC-3'	60 °C
*ID1*	5′-GGCTGTTACTCACGCCTCAAG-3'	5′-CCAACTGAAGGTCCCTGATGTAG-3'	60 °C
*NTE5*	CGCAACAATGGCACAATTAC	5′-CAGGTTTTCGGGAAAGATCA-3'	60 °C
*OCN*	5′-GTGCAGCCTTTGTGTCCAA-3'	5′-GCTCACACACCTCCCTCCT-3'	60 °C
*OSX*	5′-GAAGGGAGTGGTGGAGCCAAAC-3'	5′-ATTAGGGCAGTCGCAGGAGGAG-3'	60 °C
*RUNX2*	5′-CGCATTCCTCATCCCAGTAT-3'	5′-GCCTGGGGTCTGTAATCTGA-3'	60 °C
*SOX9*	5′-TGGAGACTTCTGAACGAGAGC-3'	5′-CGTTCTTCACCCACTTCCTC-3'	60 °C
*THY1*	5′-TCGCTCTCCTGCTAACAGTCT-3'	5′-CTCGTACTGGATGGGTGAACT-3'	60 °C
