# Supplementary material for: Bioinspired seeding of biomaterials using three dimensional microtissues induces chondrogenic stem cell differentiation and cartilage formation under growth factor free conditions
Source: Sci Rep. 2016 Nov 3;6:36011. doi: 10.1038/srep36011 (PMC5093556; doi:10.1038/srep36011)
Supplement: Supplementary Information [file srep36011-s1.pdf]

SUPPLEMENTARY INFORMATION

Bioinspired seeding of biomaterials using three dimensional microtissues induces chondrogenic stem cell differentiation and cartilage formation under growth factor free conditions

AUTHORS:

J. Leijten[1,2,3,*], L.S. Moreira Teixeira[1,2,*], J. Bolander[1,2,*], W. Ji[1,2], B. Vanspauwen[4], J. Lammertyn[4], J. Schrooten[1,2] and F.P. Luyten[1,2,#]

SUPPLEMENTAL FIGURES

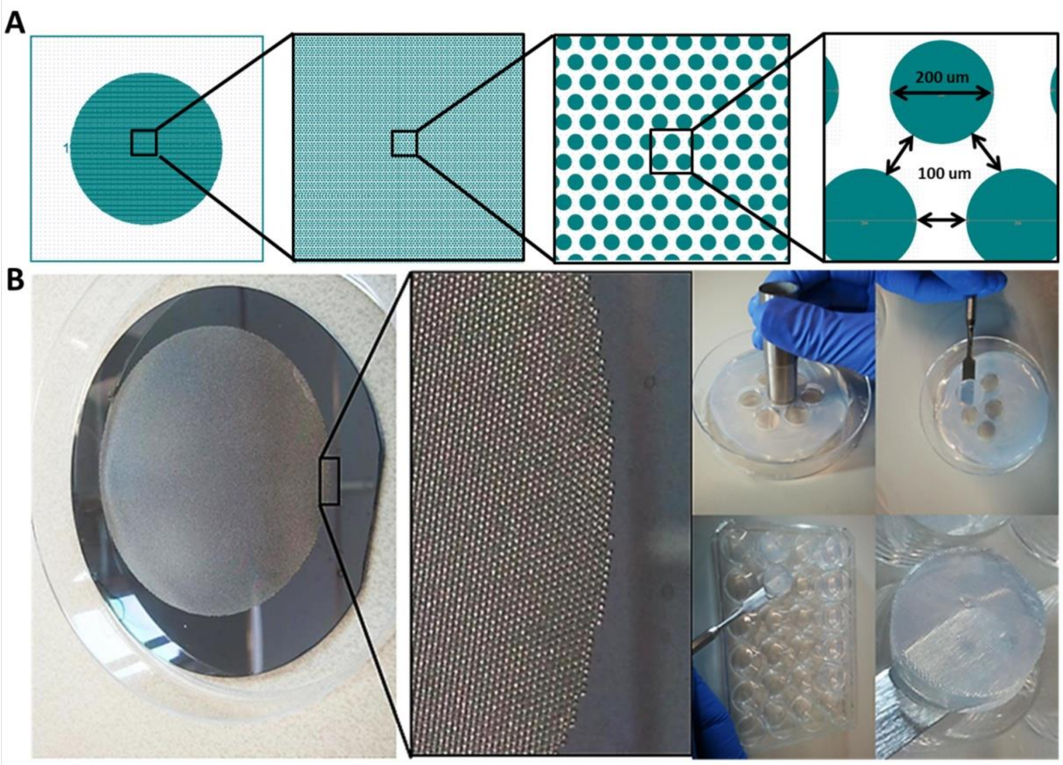

Supplemental figure 1. Design and fabrication of microaggregate forming agarose microwells. (A) A photomask was designed containing a circular array of 125.000 circular spots. Each spot has a radius of 200 micrometer and is spaced minimally 100 micrometer from each other spot. (B) The patterned silicon wafer was used to create 3% agarose negative copies. Using a sterile biopsy punchers microwell inserts were cut to tightly fit into 24 well culture plates.

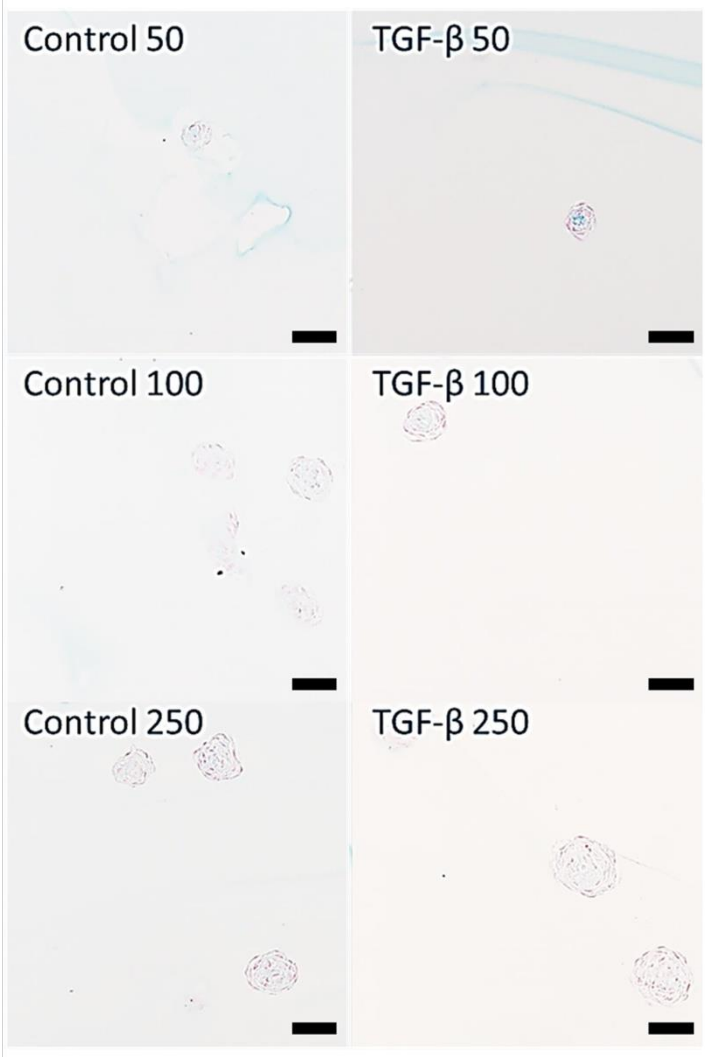

Supplemental figure 2. Implanted constructs did not demonstrate detectable levels of glycosaminoglycan. Prior to implantation, hPDC were cultured in vitro as single cells or microaggregate supplemented with 10 ng/ml of TGFB for 6 days. Specimen were fixated and histologically analyzed on glycosaminoglycan deposition using Alcian blue staining. Scale bars equal 200 μm.
